# Multi-Task End-to-End Self-Driving Architecture for CAV Platoons

**DOI:** 10.3390/s21041039

**Published:** 2021-02-03

**Authors:** Sebastian Huch, Aybike Ongel, Johannes Betz, Markus Lienkamp

**Affiliations:** 1 TUMCREATE, 1 CREATE Way, #10-02 CREATE Tower, Singapore 138602, Singapore; aybike.ongel@tum-create.edu.sg (A.O.); lienkamp@ftm.mw.tum.de (M.L.); 2 Institute of Automotive Technology, Technical University of Munich, Boltzmannstr. 15, 85748 Munich, Germany; 3 Department of Electrical and Systems Engineering, School of Engineering and Applied Science, University of Pennsylvania, 220 South 33rd Street Philadelphia, Philadelphia, PA 19104, USA; joebetz@seas.upenn.edu

**Keywords:** connected and autonomous vehicles, artificial neural networks, end-to-end learning, multi-task learning, urban vehicle platooning, simulation

## Abstract

Connected and autonomous vehicles (CAVs) could reduce emissions, increase road safety, and enhance ride comfort. Multiple CAVs can form a CAV platoon with a close inter-vehicle distance, which can further improve energy efficiency, save space, and reduce travel time. To date, there have been few detailed studies of self-driving algorithms for CAV platoons in urban areas. In this paper, we therefore propose a self-driving architecture combining the sensing, planning, and control for CAV platoons in an end-to-end fashion. Our multi-task model can switch between two tasks to drive either the leading or following vehicle in the platoon. The architecture is based on an end-to-end deep learning approach and predicts the control commands, i.e., steering and throttle/brake, with a single neural network. The inputs for this network are images from a front-facing camera, enhanced by information transmitted via vehicle-to-vehicle (V2V) communication. The model is trained with data captured in a simulated urban environment with dynamic traffic. We compare our approach with different concepts used in the state-of-the-art end-to-end self-driving research, such as the implementation of recurrent neural networks or transfer learning. Experiments in the simulation were conducted to test the model in different urban environments. A CAV platoon consisting of two vehicles, each controlled by an instance of the network, completed on average 67% of the predefined point-to-point routes in the training environment and 40% in a never-seen-before environment. Using V2V communication, our approach eliminates casual confusion for the following vehicle, which is a known limitation of end-to-end self-driving.

## 1. Introduction

One major trend in intelligent transportation systems is the development of connected and autonomous vehicles (CAVs), which has seen vast progress over the past few years. However, research on autonomous vehicles has a long history, starting in the 1980s with the PROMETHEUS project and Autonomous Land Vehicle in a Neural Network (ALVINN), an experimental vehicle that made use of neural networks for the driving task. Over 30 years later, vehicles with lower levels of driving automation—up to Level 2 as specified by SAE J3016 [1]—have become commercially available. Researchers and the industry now focus on the development of vehicles with higher levels of driving automation, such as Levels 4 and 5. These enable autonomous driving without the need for human interaction. Furthermore, highly automated vehicles can increase road safety and reduce emissions [2]. Higher levels of driving automation also allow vehicles to drive in a platoon and reduce the inter-vehicle distance. A platoon generally consists of a leading vehicle with one or more followers. The leading vehicle is driven autonomously or manually, while the following vehicle usually acts autonomously, especially at close inter-vehicle distances. Driving with a close inter-vehicle distance not only has the potential to reduce emissions on highways because of the reduction in drag and hence energy consumption, but also saves space in an already limited urban area [3,4]. For example, CAV platoons on a dedicated lane can reduce the reverse-accordion effect when jointly accelerating at a green traffic light. In this work, we focus on CAV platoons deployable in an urban area.

Key to the success of CAVs are the recent advances in machine learning (ML). Different ML techniques, e.g., deep learning, facilitate the extraction of information from a large amount of sensor data to understand the CAV’s environment. Although there exist different approaches to achieve the ultimate goal of developing self-driving cars, most of them rely on the usage of ML methods to a high degree. These approaches can be classified into the following three categories: modular pipelines, direct perception, and end-to-end deep learning.

The approach primarily used for self-driving cars employs modular pipelines and is based on the mediated-perception principle [5]. This approach makes use of the decomposition of the driving task to split the complex task into successive modules (perception, prediction, planning, and control) [6]. Each module consists of a range of specialized submodules. Using onboard sensors like the camera, LiDAR, radar, GNSS, and the inertial measurement unit, the perception module creates a comprehensive internal representation of the self-driving car’s environment. Given the internal environment’s representation, the planning module is responsible for the route, path, and motion planning [7]. The subsequent control module converts the trajectories generated by the planning system into commands for the self-driving car’s actuators of the steering wheel, engine, and brakes.

Although the modular pipeline approach has good interpretability, the information computed at every time step adds unnecessary complexity to the system. Additionally, a large amount of labeled data (e.g., 3D bounding boxes [8] or pixel-wise semantic segmentation [9]) are needed for supervised training of the submodules of the perception module. These data are costly and hard to obtain. Additionally, some localization algorithms require accurate high-definition road maps [10], which have to be generated offline. Another problem is the error propagation that can occur in multiple sections of the modular pipeline [11].

Based on the psychological theory about perception [12], the direct-perception approach was proposed by [13] to solve the self-driving challenge. Rather than splitting the entire driving task into smaller submodules, the authors used a single neural network in the perception module to predict meaningful affordances. These affordances are a low-dimensional compact and intermediate representation of the CAV’s surroundings, as opposed to the high-dimensional output of the modular pipeline approach. Examples of affordances used by the authors are the car’s angle relative to the lane, distances to lane markings, and cars in the current and adjacent lanes. Based on the affordances, a simple car controller outputs the commands to drive the vehicle. The authors proved the generalization ability of their system by testing the model and trained with data captured in the TORCS simulator, on real-driving videos. Reference [14] generalized the direct-perception approach to address the task of driving in an urban environment, which demands additional affordance indicators. A general problem posed by the direct-perception approach is that the affordances are manually chosen and may not be suitable for the complex driving task, i.e., not all situations can be covered by a few low-dimensional affordances.

The third paradigm for autonomous driving is end-to-end learning [15]. In general, the idea of end-to-end learning is to train a neural network that directly maps the raw sensor data to driving commands, i.e., it outputs the steering angle and throttle or brake values. The input data may come from different sensors, such as mono cameras, stereo cameras, LiDAR, or any combination thereof. However, the most widely used sensor is the mono camera. In recent years, convolution neural networks (CNN) [16] have been the most widely used method for feature extraction of image data, since CNNs achieved the best results for image recognition at the ImageNet Large Scale Visual Recognition Challenge (ILSVRC) [17]. The training is based on supervised learning. During the training phase, the networks process the images and the corresponding labels. During inference, the networks predict these labels, with images as the only input. In direct comparison, the driving performance of the end-to-end approach is comparable to the driving performance of the modular pipeline, but it is less fragile in new environments [18]. A benefit of this approach is that there is no need for hand-engineered heuristics, and hence, the system is more robust for unpredictable situations. Furthermore, collecting labeled training data on an image-level for end-to-end learning is easier than on a pixel-level for the modular pipeline approach.

In this work, we propose a multi-task deep learning architecture for CAV platoons that is trained in an end-to-end fashion. This architecture is based on vision only and can be used in both the leading vehicle and following vehicles to maneuver them. The vehicles communicate via V2V communication to improve the performance. The neural network was tested in a never-seen-before urban environment in a simulation under different weather conditions and with dynamic traffic. A two-vehicle platoon followed the road while obeying traffic rules, such as traffic lights at intersections. We benchmarked the performance of our approach with state-of-the-art metrics and demonstrated the capability our model taking over control of a two-vehicle platoon to complete predefined routes.

## 2. Related Work

Research on the platooning of vehicles has a long history, although it is mainly focused on the energy savings or communication topology of platooning vehicles. Preliminary work on autonomous truck platooning was undertaken by [19] in 1995. The authors tested a two-vehicle platoon with a manually-driven leading truck followed by an autonomous truck. The sensor setup for the following truck consisted of a single front-facing camera together with the vehicle states (e.g., acceleration and velocity) of the leading truck, which were obtained via V2V communication. This vision-based approach estimated the truck’s heading based on the lane markings and the distance to the leading truck by visual detection of active infrared lights on the rear of the leading truck. Major large-scale pilot studies were also conducted to determine the feasibility and benefits of truck platooning on highways. These works include the European research programs CHAUFFEUR and CHAUFFEUR2 in the early 2000s [20]. Other similar projects were the California PATH truck-platooning program and KONVOI [21]. A more comprehensive review of these projects can be found in [22].

End-to-end self-driving: The origin of this approach dates back to the late 1980s. In 1989, ALVINN [23] used a small (by today’s standards) fully connected network to predict the turn curvature in front of the car. The inputs were images captured by a camera and scans from a laser range finder. The training data were collected using a simulated road generator, which created video images, as well as laser range finder images. As a result, ALVINN was capable of following a 400 m section of the road at a speed of 0.5 m/s. Instead of a fully connected network, Reference [24] implemented a six layer CNN in the remote-controlled mobile robot DAVE. DAVE was intended to operate in unknown off-road terrain and avoid smaller obstacles such as trees and ponds. The input of this network consisted of images from a front-facing stereo camera, and the output was the steering angle. The network was trained with images and control commands recorded while a human was maneuvering the robot in different terrains and light conditions. After the training, DAVE managed to drive approximately 20 m on average until it hit an obstacle [25]. Reference [26] used the TORCS simulator to train an agent to drive on a race track by employing reinforcement learning. This approach is solely vision-based, with a recurrent neural network receiving front-facing images and predicting the steering angle, brake, and throttle. The performance is comparable to that of hard-coded controllers, which have access to all vehicle states. A seminal work on end-to-end learning is DAVE-2 [25]. In 2016, the authors designed and trained a CNN named PilotNet to steer a full-sized vehicle. Unlike the mobile robot DAVE, which was tested a decade earlier, DAVE-2 was trained with about 72 h of real-world driving data, captured by three front-facing cameras while driving on public roads under different light and weather conditions. The network training benefited from two off-center cameras, which implied a shift from the center of the lane and thereby enabled the network to learn to recover from mistakes. Only the center camera was used for testing.

Similar systems have been tested in recent years, with training data either taken from real-world datasets [27,28,29,30,31,32] or generated in simulations [33,34,35,36]. Some of this research explored the benefits of including temporal dependencies between consecutive images by adding recurrent layers to the network. For example, References [27,30,31] added long short-term memory (LSTM) layers on top of the CNN. The CNN was responsible for feature extraction of the images, while the LSTM incorporated features of the last time steps for the driving decision of the current time step. Reference [36] followed the same approach and additionally implemented the end-to-end approach in a simulated urban environment. To obey the traffic rules, the authors added additional inputs to the network, such as the relevant traffic light state and speed limit. Application of end-to-end self-driving to vehicle platooning: The above works did not address end-to-end deep learning algorithms that are specifically designed for the application in vehicle platoons. This topic was first addressed in [32,34].

Reference [34] used the end-to-end learning of CNNs to maneuver a truck behind a leading truck in a simulation based on vision only. The output of the network was branched and predicted the steering angle and throttle/brake. The simulation environment was a flat terrain in an off-road area. The leading truck made random driving decisions, while the following truck was operated manually during data collection. The labels associated with each image consisted of discrete steer and throttle/brake values, making the training a classification problem. After supervised training, several test runs were conducted. Although this was the first work to address end-to-end self-driving for CAV platoons, it did not consider an urban environment and focused on the following vehicle only. Since the leading vehicle was manually driven, the platoon could not operate fully autonomously. Furthermore, the system setup produced discrete output values that would not allow smooth driving behavior. Additionally, because the network was tested in the same simulation environment that it was trained in, its ability to generalize and extrapolate data to unseen environments was not assessed.

In 2019, Reference [32] was the first to mention the additional implementation of V2V communication to enhance the performance of end-to-end self-driving. The authors proposed a network architecture that processed images recorded by two cooperative self-driving vehicles simultaneously. The goal was to predict the steering angle of the following vehicle for lane-keeping on highways. Via V2V communication, the image data from a leading vehicle were transmitted to the following vehicle, where they were merged with the follower’s images. The authors argued that adding images from the leading vehicle improved the performance of the system because there was more available information. Using information from the leading vehicle can improve driving behavior, but the image stream between the cars as proposed by the authors transmits information at a high level. Simple low-level vehicle states, e.g., acceleration and velocity, are easier to transmit because of the reduced data size. Moreover, the images of the leading vehicle might already be processed by the network of the leading vehicle itself, making a second processing in the following car redundant.

With our work, we want to address the limitations of [32,34]. Our contributions are the implementation of an end-to-end self-driving model that:

- is not limited for deployment in following vehicles, but can also be used in leading vehicles of a CAV platoon, hence performing multiple tasks;
- is predicting continuous instead of discrete control commands for a smooth driving behavior;
- is not limited to operating in off-road or highway environments, but can be used in a challenging urban environment;
- and is using V2V communication, but transmitting low-level vehicle states instead of a high-level image stream.

## 3. Models

In this section, we introduce our proposed architecture for CAV platoons and modified architectures of this baseline for comparison. Before going into the details of the architectures in the following subsections, some features that all models have in common are discussed. The last part of this section addresses the specifics of training that come along with multi-task learning.

The general vision-based end-to-end approach for CAVs maps observations $s_{t}$, e.g., images, at each time step *t* to an action $a_{t}$. This implies that there exists a driving policy that satisfies a function *F*, which describes the relationship between $s_{t}$ and $a_{t}$, that is $a_{t}=F\left(s_{t}\right)$. Given a dataset $D={\left\{\left(s_{i},a_{i}\right)\right\}}_{i=1}^{N}$ with expert driving demonstrations, the goal of the network training process is to optimize the parameters θ of a function G(s,θ) to approximate $F(s_{t})$ by minimizing the loss L:

(1) $$\min_{\theta }\sum_{i}L\left(G\left(s_{i},\theta \right),a_{i}\right).$$

While the observations $s_{t}$ usually contain images, they can be extended by including vehicle states or other measurements.

To operate a CAV platoon, at least two vehicles are controlled simultaneously with slightly different tasks. The leading vehicle follows a route while staying in the lane, obeying traffic rules and avoiding collisions with static and dynamic objects. It makes driving decisions based on the perceived environment and the high-level plan (e.g., navigation to a predefined destination). Following vehicles in a platoon, however, have limited front visibility because of their close proximity to the preceding car. Furthermore, as long as they are part of the platoon, they follow their immediate predecessor while staying in the lane. Following a preceding vehicle can be further simplified for a homogeneous CAV platoon, in which all vehicles are identical. In this case, any following vehicle of the platoon follows a preceding vehicle with similar visual features to all other vehicles, independent of its position.

The different tasks of leading and following vehicles have different requirements for the inputs and outputs of the network. The basic input for the multi-task network in both vehicles is images from a single front-facing camera. Other sensor modalities are not taken into account to avoid sensor fusion. We chose a single camera over stereo cameras to overcome the calibration problem. When used in the leading vehicle, two additional inputs are added to the network, that is the current vehicle speed and the high-level command. The current vehicle speed is added as an input since it influences the longitudinal driving decisions [37]. The high-level command provides information about the driving direction. Possible discrete states are 〈*Left, Straight, Right, No Intersection*〉. The state *No Intersection* is active when the leading vehicle is not in close proximity to an intersection, whereas the other three states indicate the driving direction at an upcoming intersection. These states can be provided by a high-level planner, e.g., a navigation system. As stated before, the following vehicle always follows the leading vehicle, and therefore, the velocity and high-level command inputs are not required.

The outputs for the leading vehicle are the control commands, including steering angle and throttle/brake. For the following vehicle, the outputs are the steering angle and gap between the vehicles. We did not choose the throttle/brake as the output for the following vehicle because of the ambiguity of the images, known as casual confusion [38]. The images showing an acceleration and deceleration phase may look similar, but have different labels, and hence, the network cannot distinguish between these situations. To overcome the casual confusion for the following vehicle, we chose to predict the gap between the cars, which is unique for each image. This can further improve the steering-angle prediction, since the network learns to focus on the leading vehicle during training.

We combined the driving task for the leader and follower into a single multi-task network, which is based on the following constraints. First, we used identical vehicles as the leader and follower, so that the camera position in both vehicles was the same. Second, the shared layers perceived a similar visual input, namely the image of the environment in front of the vehicle. We argue that each task can benefit from the other: the leading vehicle trains the network to focus on the road and lanes in general, while the following vehicle strengthens the network to detect dynamic objects. Additionally, the joint training with different datasets can improve the generalization ability of the network.

The information flow topology of the V2V communication follows the predecessor-following principle as described in [39], in which following vehicles in the platoon receive information from their immediate predecessor. The control of following vehicles is a combination of a neural network for the lateral control and a conventional car-following model for the longitudinal control. The car-following model follows a simple constant spacing control strategy, which is sufficient for our experiments (ensures weak string stability), but it could be replaced with different control strategies.

### 3.1. Model A: Multi-Task Network Baseline

**Figure 1:**
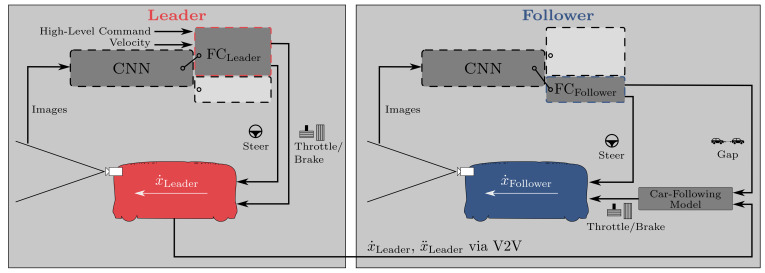
Overview of the proposed system. The leading and following vehicle use the same multi-task network architecture (for details, see Figure 2), but activate different prediction heads. The acceleration and velocity of the leading vehicle are transmitted to the following vehicle via V2V. Details on the car-following model used in the following vehicle can be found in (2).

Our proposed multi-task network consists of a shared CNN acting as a feature extractor and two prediction heads (fully connected (FC) networks) for the leader or follower, respectively. As illustrated in Figure 1, the same trained network can be used for the leading or following vehicle. Different inputs and outputs are activated or deactivated, depending on in which vehicle the network is used. In the leading vehicle, the network takes images as the input for the CNN. Together with the high-level command and the velocity, the output of the CNN is processed in an FC network to predict the steering angle and throttle/brake, which are directly applied as the vehicle controls. In the following vehicle, the network also uses images as the input for the CNN. The subsequent FC network predicts the steering angle and the gap between the vehicles. As in the leading vehicle, the steering angle is directly used for vehicle control. The predicted gap, together with the acceleration and velocity of the leading vehicle (transmitted via V2V communication), serves as the input for a simple car-following model, which is similar to [40]. The desired acceleration ${\ddot{x}}_{\mathrm{Follower}}\left(t\right)$ of the vehicle is calculated based on the acceleration of the leading vehicle ${\ddot{x}}_{\mathrm{Leader}}\left(t\right)$, the velocity difference between both vehicles $\Delta \dot{x}\left(t\right)$, the predicted gap s(t), and the desired gap $s_{\mathrm{des}}$. The influence of the leader’s acceleration, the velocity difference, and the gap is tuned with α, β, and γ, respectively (α=1.0, β=0.75, and γ=0.2 in our experiments, similar to [40]). As with [41], α=1.0 guarantees weak string stability for this constant spacing control strategy.

(2) $${\ddot{x}}_{\mathrm{Follower}}\left(t\right)=\alpha {\ddot{x}}_{\mathrm{Leader}}\left(t\right)+\beta \Delta \dot{x}\left(t\right)+\gamma \left(s\left(t\right)-s_{\mathrm{des}}\right)$$

**Figure 2:**
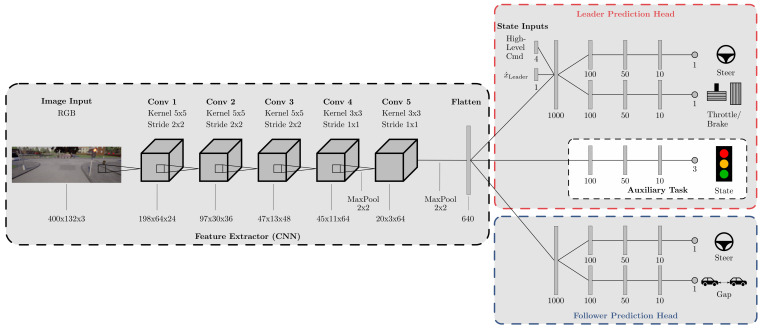
Detailed network architecture. The proposed multi-task deep learning network for connected and autonomous vehicle (CAV) platoons (Model A) with the details of the architecture. The feature extractor is a CNN based on PilotNet [25]. Following the feature extractor, the network consists of two prediction heads to serve its tasks in the leading and following vehicle, respectively. The prediction heads consist of FC networks with multiple branches for the individual outputs.

The detailed multi-task neural-network architecture is shown in Figure 2. The feature extractor is similar to the CNN of PilotNet [25]; however, two additional max pooling layers are inserted after the last two convolutional layers to reduce the dimensions. The input of the feature extractor is RGB images with 400×132 pixels normalized to [−1,1]. The feature extractor output is shared by the prediction heads of the leader and the follower.

The prediction head for the leader is split into two branches predicting the steering angle and the throttle/brake. These two branches include the additional inputs, namely the high-level command and the ego velocity. The inputs are concatenated with the feature extractor output and jointly serve as the input for the subsequent shared fully connected layer with 1000 neurons. An auxiliary classification task is added within the leader head to classify the status of a traffic light within sight. This auxiliary task is activated only during training and helps the network to focus on traffic lights to improve the throttle/brake prediction. We define three different output states 〈*no traffic light, red, green*〉. The state *no traffic light* is active if there is no traffic light within 30 m along the path. Below 30 m, the auxiliary task classifies the state of the traffic light as either *red* or *green*. The auxiliary task is directly connected to the feature extractor output without the influence of the additional inputs. This way, the CNN learns to predict the traffic light state using visual clues only. The prediction head of the follower is also split into two branches for the prediction of steering angle and vehicle gap.

The structure of all branches was inspired by the FC network of [25]. We use fully connected layers with a decreasing number of neurons at each subsequent layer. All layers use dropout to improve the generalization of the network, with a dropout probability of 0.2 for convolutional layers and 0.4 for fully connected layers (at each batch, we ignore 20% and 40% of the neurons per layer, respectively). The activation function for all layers except the output layers is the rectified linear unit (ReLU). The steering and throttle/brake output layers use tanh (limits outputs to (−1,1)); the traffic light output (auxiliary task) uses softmax for classification (outputs class probabilities); and the gap prediction uses a linear activation function for regression. Contrary to [34], the outputs for the throttle/brake and steering are continuous values instead of discrete values, which results in more realistic and smoother driving. In total, the baseline model has 1,910,747 trainable parameters. This relatively small number of parameters may help to reduce overfitting and decreases training time.

### 3.2. Model B: Multi-Task Network with LSTM Extension

Model B seizes the idea of [32] to incorporate time-distributed images using a recurrent neural network and is implemented to compare this approach with Model A. Different from [32], V2V communication is not used to send an image stream, but to transmit low-level vehicle states as in Model A. Furthermore, as [32] was limited to lateral vehicle control, the outputs of Model B are identical to the outputs of Model A for lateral and longitudinal control.

We add three LSTM layers between the feature extractor and the prediction heads. Each LSTM layer has 128 neurons to keep the overall parameter count comparable to that of Model A (multi-task network baseline). In total, the multi-task network with the LSTM extension has 1,492,443 parameters. The activation function of the LSTM layers is tanh, and the weights are initialized with the Xavier initialization (most widely used for the tanh activation function). We do not use dropout for the LSTM layers.

**Figure 3:**
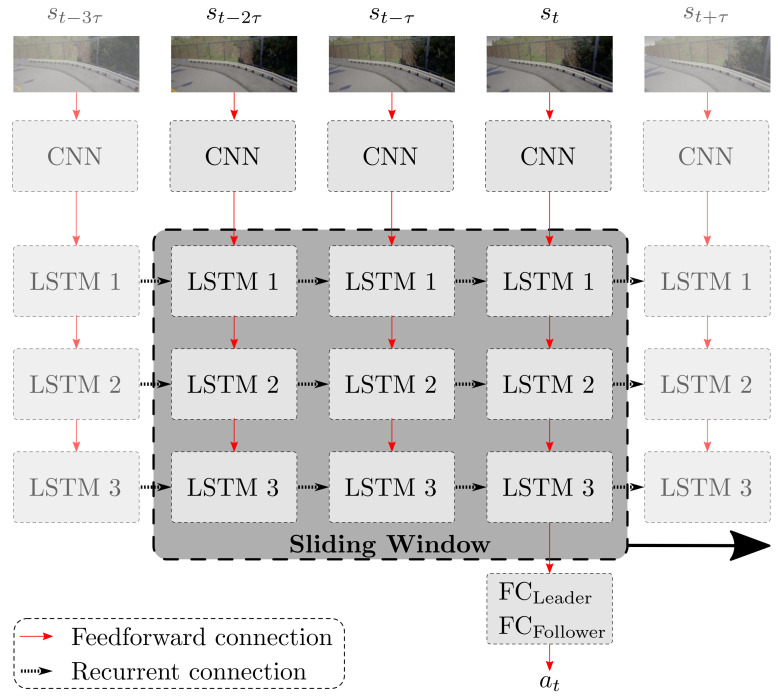
Model B: multi-task network with LSTM extension. Three LSTM layers follow the feature extractor output. The sliding window method includes the last *ℓ* images with a time step of τ between the images.

The LSTM network has two variables, namely the sequence length and the sampling interval. The sequence length *ℓ* defines the number of images from the past, including the image at the current time step, which are used to make the prediction at the current time step. The sampling interval τ describes the number of steps between the consecutive images used in the LSTM network. For example, a sequence length of ℓ=5 combined with a sampling interval of τ=2 takes every other image of the training data five times in order to fill one sequence. Therefore, this exemplary sequence covers a period of nine consecutive images. We use a sliding window to generate the sequences during training, following [30]. Instead of using fixed consecutive sequences, the sliding window ensures that, within a training epoch, every image is used once at all positions of the sequence. The principle of the sliding window with a sequence length of three is depicted in Figure 3.

### 3.3. Model C: Multi-Task Network with Pre-Trained Feature Extractor

Instead of training a network from scratch, transfer learning can be applied to use pre-trained networks. The idea behind transfer learning is that a network trained on a large dataset, such as a dataset for object detection, can be applied in a different domain. We replace the feature extractor (PilotNet CNN) in our architecture with a CNN with more convolutional layers; in particular, we use ResNet-50 [42] pre-trained on the ImageNet (ILSVRC) dataset. To adapt this network to our existing architecture, we remove the last classification layer of ResNet-50 and replace it with a max pooling layer. This last layer serves as the input for the prediction heads. The prediction heads are identical to the prediction heads of Model A. The weights of the layers in the first seven of 16 residual blocks are not trainable and are therefore blocked from updating to retain the lower-level features. Embedded in our architecture, the complete network has 28,300,999 parameters.

### 3.4. Model D: Single-Task Networks

To compare the multi-task network baseline with single-task networks, we split our multi-task network into two single-task networks for the leading and following vehicle. Each network is trained separately with the corresponding part of the dataset. These models should provide information on whether multi-task learning is beneficial for the driving performance. The single-task network for the follower is similar to the model of [34] and serves as a comparison with their approach. Different from [34], Model D uses continuous control commands and V2V communication identical to Model A.

### 3.5. Model E: Multi-Task Network without Auxiliary Task

The auxiliary task is added to the multi-task network to raise the network’s awareness for traffic lights during training. In Model E, the auxiliary task is removed, while the rest of Model E is identical to the multi-task network baseline (Model A). This model is used in a separate experiment to provide information on whether the auxiliary task leads to a performance increase of the multi-task network.

### 3.6. Multi-Task Network Training

The multi-task networks are trained in single supervised training runs, where the training data contain images from both the leading and following vehicle. This has advantages over successive training, as in the latter, the network is prone to developing a bias towards the data with which it was initially trained. To jointly train the network to achieve good performance for both tasks (i.e., in the leading and following vehicle), we use a dynamically weighted loss function,

(3) $$L=\left(1-\eta \right)L_{\mathrm{Leader}}+\eta L_{\mathrm{Follower}}$$

where η weights the loss functions of the leader and follower prediction heads. For each batch, we train with either $L_{\mathrm{Leader}}$ or $L_{\mathrm{Follower}}$, i.e., a batch never consists of samples from both a leading and a following vehicle. If a batch consists of samples only showing images captured by the leading vehicle, we set η=0, otherwise η=1. This way, we can control in which prediction head the weights are updated during gradient backpropagation.

The leader and follower loss functions are summations of the individual loss functions of each branch within the prediction heads. For the leader loss function, that is:

(4) $$L_{\mathrm{Leader}}=\sum_{i=1}^{3}\lambda_{\mathrm{Leader}}^{i}L_{\mathrm{Leader}}^{i}$$

with the two individual loss functions for the two branches of steering and throttle/brake and the auxiliary loss for the traffic light classification. The factor $\lambda_{\mathrm{Leader}}^{i}$ weights the individual and auxiliary loss functions. Similarly, the follower loss function is defined as:

(5) $$L_{\mathrm{Follower}}=\sum_{i=1}^{2}\lambda_{\mathrm{Follower}}^{i}L_{\mathrm{Follower}}^{i}$$

with the two individual loss functions for steering and gap. We use the mean squared error for all individual loss functions except for the auxiliary loss $L_{\mathrm{Leader}}^{\mathrm{Traffic}\mathrm{Light}}$, where we use the categorical cross-entropy since it is a classification output.

## 4. Experimental Setup

This section describes the simulation environment, the training dataset, the training process, and the experiments conducted for evaluation.

### 4.1. Simulation Environment

For training and validation data, we used data generated in a simulator rather than real-world datasets. This was because we needed a special dataset containing vehicle platooning scenarios, which is not available in common datasets. The simulator also allowed us to test the performance of the networks in different environments, compared with only using a subset of the dataset as test data. We recorded the dataset in the CARLA simulator [18], which provides different environments with rural or urban landscapes and allows the configuration of environmental conditions such as weather or light.

**Figure 4:**
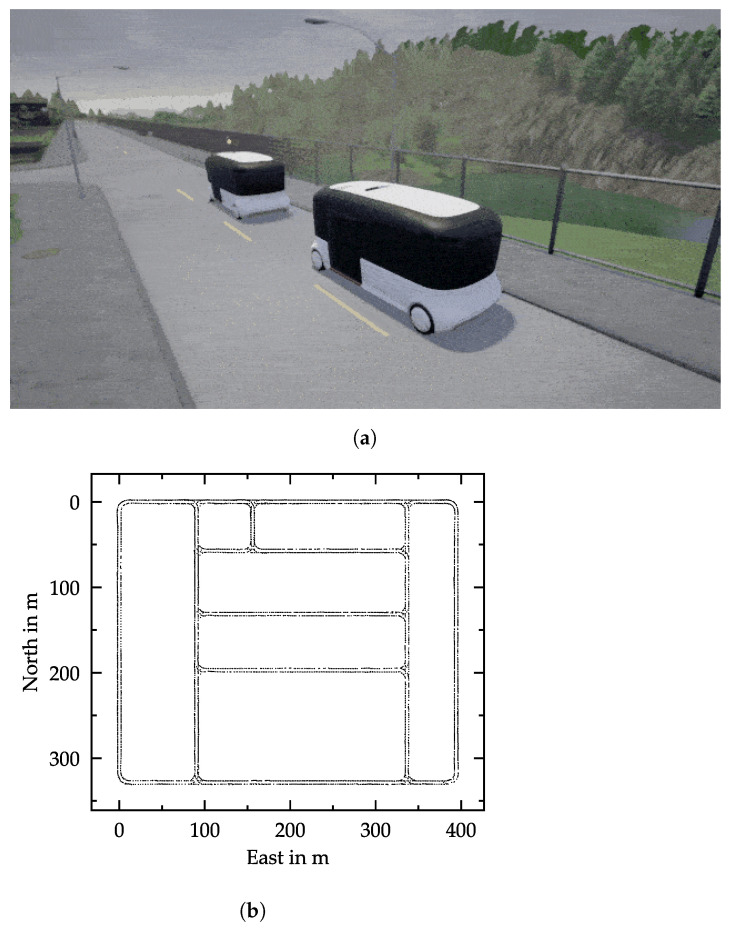
Simulation environment. (**a**) Two-vehicle platoon with minibuses used for dataset recording in the CARLA simulator [18]. (**b**) Map of *Town01*, which serves as the urban environment for the training and validation dataset recording. Each point represents a position visited by the vehicles during dataset recording. Smaller deviations from the center line are due to injected noise (lateral shifts).

Our experimental vehicles in the simulation were minibuses with a length of 6 m; see Figure 4a. The specifications of these minibuses are similar to 2getthere’s GRT [43] or Navya’s Autonom Shuttle Evo [44]. Each vehicle was equipped with three front-facing cameras: a center camera at a height of 2.7 m and two cameras located at the left and right at a distance of 0.5 m from the center camera. Only the center camera was used during testing. The off-center cameras simulated a shift from the center of the lane and improved the ability of the network to recover from disturbances, as with [25]. The image resolution of each camera was 400×132 pixels with a field of view of $100^{\circ }$ and a pitch of $-15^{\circ }$. We recorded at 10 fps, as a higher sample rate would produce more similar images.

During the dataset recording, both vehicles of the platoon drove autonomously and randomly in the simulated urban environment, capturing images and their corresponding labels. Both vehicles used a Stanley controller for the lateral control (minimizing heading error and cross-track error). The gap between both vehicles was varied to capture a broad range of gaps. The leading vehicle obeyed traffic rules such as traffic lights and speed limits and paid attention to other road users. The following vehicle followed the leader all the time. We injected noise into both vehicles by occasionally shifting them laterally. This happened every 10 to 15 s with a probability of 23. The training data were captured in CARLA *Town01*, and testing was conducted in *Town01* and *Town02*. *Town01* (see the map in Figure 4b) has 2.9 km of drivable roads in total, and *Town02* has 1.4 km of drivable roads [18]. We used several weather and light settings during training.

### 4.2. Training Dataset

In total, we collected 160,000 images from both the leading and following vehicles, which corresponds to about 4.5 h or 75 km of driving. We split our dataset into a training part and validation part. The validation part was used to monitor the validation loss during training and consisted of 10,000 samples, and the remaining 150,000 samples were used for training. The 10,000 samples of the validation part were the last 6.25% of the complete dataset, a similar ratio to [14]. As with [45], we did not randomly select samples for the validation dataset from the complete dataset, as this would have led to the validation dataset being similar to the training dataset. The testing was directly conducted in the simulation, so we did not need a testing dataset.

**Figure 5:**
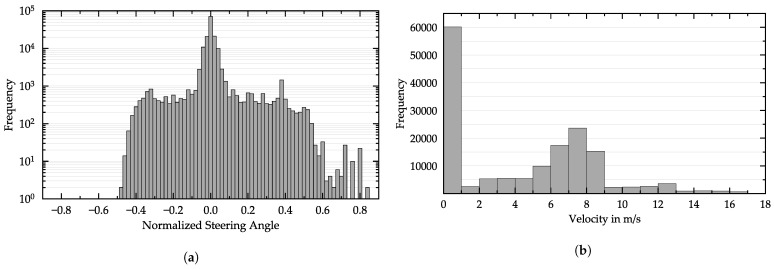
Distribution of the captured dataset for (**a**) the steering angle and (**b**) the velocity. The dataset contains 160,000 samples in total.

The majority of the training data consisted of images showing the vehicle driving straight, as seen in the histogram in Figure 5a. The higher the steering angle was, the fewer training samples were in the dataset. Note the asymmetry with more samples showing higher right steering angles (positive) than left steering angles (negative). This is because of the right-hand traffic in the simulation, which usually leads to higher steering angles for right turns than for left turns. Training with a dataset containing mainly steering angles around zero degrees could have led to a network that was biased towards driving straight. To compensate for this, we upsampled images showing higher steering angles. We selected a steering angle threshold of $\pm 20^{\circ }$, and images satisfying this threshold were upsampled with a factor of three. Many samples of the training dataset showed the vehicle waiting at a red traffic light with a velocity of 0 m/s (Figure 5b). While staying static at a red traffic light, the images were nearly identical. For this reason, we downsampled these images by reducing their occurrence by 50%.

In the dataset, the actions for every state are the steering and throttle/brake values present at the moment the state, i.e., the image, was captured. However, during testing, the network should predict the control commands for the next discrete time step, since the current time step was already executed. For this reason, we shifted all actions to the previous state, i.e., an image captured at time *t* is labeled with the control commands that were applied at the next time step t+1.

### 4.3. Network Training

The goal of the training was to minimize the dynamic weighted loss function described in (3). We used the AdaMax optimizer with an initial learning rate of $10^{-4}$. However, the learning rate was reduced when the training stagnated. We tried different initial learning rates from $10^{-3}$ to $10^{-6}$, but achieved the best results with $10^{-4}$. Network weights were initialized with the He or Xavier initialization for layers with ReLU or tanh activation functions, respectively. The performance decreased with other weight initialization techniques (e.g., He initialization with a normal distribution instead of a uniform distribution). Training data normalization was performed for all input and output data. The images, steering angle, and throttle/brake were normalized to [−1,1], and the velocity and the gap were normalized to [0,1]. The high-level command was encoded in a one-hot vector, and we used a batch size of 128 samples. Larger batch sizes not only require more memory, but also can lead to a lower generalization ability [46].

Prior to feeding the data into the network, we augmented the images with random rotations, shifts, zooming, and brightness changes in the following ranges, each with a continuous uniform distribution:

- Rotation in the range $\left[-5^{\circ }\;...\;5^{\circ }\right]$
- Horizontal shift in the range [−5%...5%]
- Vertical shift in the range [−5%...5%]
- Zoom in the range [−10%...0%]
- Brightness change in the range [−10%...10%]

**Figure 6:**
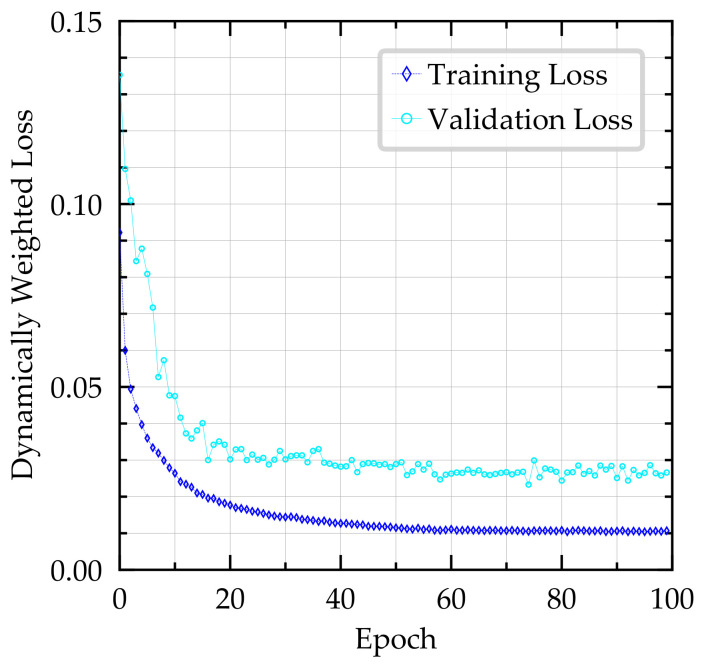
Dynamically weighted loss curve for Model A. The validation loss is calculated at the end of every epoch. Note that for this model, the lowest validation loss was achieved at Epoch 74.

During training, we observed the training and validation loss of the joint and individual loss functions. We saved the model after each training epoch and stopped the training as the loss converged after about 100 epochs (∼27 h of training time), as shown in Figure 6 for Model A. We used the model with the lowest validation loss for testing, which was not necessarily the last model.

### 4.4. Experiments and Evaluation Metrics

We conducted two experiments to evaluate the performance of the multi-task network: predefined point-to-point routes and free driving. In both experiments, each trained Model A–D was tested in three different scenarios separately. First, we assessed the performance of the model taking over control of the leading vehicle, without considering any following vehicles. In the second scenario, the model took over control of only the following vehicle, while the leading vehicle was controlled by the controller of the simulation. Finally, we evaluated the performance of a two-vehicle platoon in which two instances of the model took over the vehicle control of both vehicles. In this scenario, an episode ends if any vehicle could not complete the objective due to a crash.

The models were tested in two different environments in the simulation, a similar arrangement to that for the tests conducted by [14,35,36]. The first environment was the same as the one in which the training data were collected (*Town01*). Thus, the network had already processed similar images during training. The second environment was a never-seen-before map with an urban landscape (*Town02*). This environment was used to analyze the generalization ability of the network for unseen places.

Point-to-point routes: As with the test scheme of [18], we used point-to-point routes where the vehicle had to make several turns and drive through intersections to reach its destination. Models A–D were tested on 25 randomly generated routes, with each route repeated twice, which gave a total of 50 episodes per tested model. The length of each route was 500 m, with zero to eight signalized intersections per route. An episode was finished as soon as the vehicle reached the destination or hit an obstacle. The average completion rate (ACR) of the episodes was reported for every model.

Free driving: In addition to the average route completion, we recorded the mean time to failure (MTTF) for all models, as with [34]. A failure was a crash with an object, a stop longer than 60 s, or a high-level command not being obeyed. Traffic light violations did not count as failures, but were recorded for a separate evaluation. For the calculation of this metric, we placed the vehicles in the simulated environment and let them drive randomly. At intersections, the high-level command was chosen randomly based on the intersection layout. If a crash occurred, the episode was stopped and the time to failure was logged. The MTTF was calculated by taking the average of the individual TTFs of 10 episodes per model and scenario. The maximum TTF per episode was limited to 1440 s, and exceeding this limit triggered a new episode. This setup was also used to assess the impact of the auxiliary task, via calculation and comparison of the average violation rate of red traffic lights (AVR) for Model A and Model E. The AVR is the sum of red traffic light violations divided by the total number of traffic lights passed.

## 5. Results

**Figure 7:**
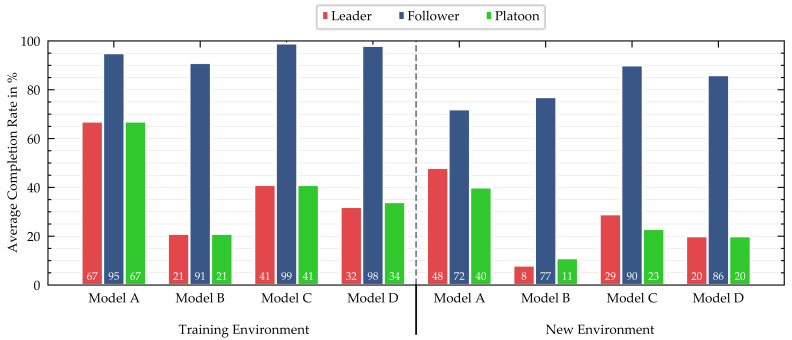
Average completion rate of the routes in % for four different models in two environments. The results for the platoon refer to a two-vehicle platoon consisting of one leading and one following vehicle (**A**: multi-task network baseline, **B**: multi-task network with LSTM extension, **C**: multi-task network with pre-trained feature extractor, **D**: single-task networks).

Point-to-point routes: The results of the quantitative evaluation of the point-to-point routes with a static environment are summarized in Figure 7. The graph shows the average completion rate of the point-to-point routes for Models A–D. The results for the platoon refer to a two-vehicle platoon consisting of one leading and one following vehicle. In general, all models performed better in the training environment than the never-seen-before environment, which was to be expected.

The multi-task network baseline (Model A) achieved the highest ACRs in the vehicle platoon and leader scenario in both simulation environments. The performance of the two-vehicle platoon is limited by the leading vehicle’s performance, which is reflected by similar results for leader and platoon. Whenever the following vehicle could not complete an episode, it was in all cases the result of a crash into static objects. However, we observed that, in 35% of the uncompleted episodes for the leader, the vehicle did not accelerate after a full stop or directly at the beginning of an episode. This is due to the casual confusion, as shown in [47]. We could solve the casual confusion for the following vehicle by implementing V2V communication, but it still persisted for the leading vehicle.

The LSTM extension (Model B), similar to [32], achieved a performance similar to that of Model A for the following vehicle. We observed low-frequency lateral oscillations of both vehicles within the lane. These oscillations led to the vehicles leaving the lane and resulted in crashes in some cases. The casual confusion was more severe than with Model A and in many cases caused zero acceleration at the beginning of an episode.

Replacing the feature extractor with the pre-trained ResNet-50 (Model C) led to superior performance of the following vehicle and driving that was nearly without failures. Although the driving performance of the leading vehicle was good, we observed that this model did not always follow the high-level command.

Even though the single-task network (Model D) of the leader did not share the feature extractor with the follower and therefore could specialize the feature extractor to its sole needs, the performance was worse compared with the multi-task network baseline.

We tested the driving behavior of the multi-task network baseline (Model A) with dynamic traffic. The performance of both the leader and follower decreased mainly as a result of crashes with other vehicles, with ACRs of 57 and 72%, respectively, in the training environment. Casual confusion after stopping behind other vehicles was another reason for the reduction in the leader’s performance.

**Figure 8:**
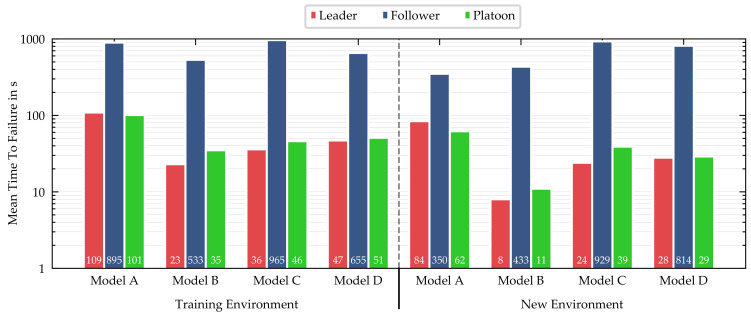
Mean time to failure (MTTF) in seconds for four different models in two environments. The results for the platoon refer to a two-vehicle platoon consisting of one leading and one following vehicle (**A**: multi-task network baseline, **B**: multi-task network with LSTM extension, **C**: multi-task network with pre-trained feature extractor, **D**: single-task networks).

Free driving: The MTTF results are summarized in Figure 8, separated into model (A–D), scenario (leader, follower, platoon), and environment. In case the failure was caused by casual confusion, the TTF was taken at the moment the vehicle stopped. The MTTF results confirm the ACR results of the point-to-point routes, with minor exceptions. We limited the maximum TTF per episode to 1440 s; however, the longest episode without failure for the following vehicle lasted close to one hour and covered nearly 14 km of autonomous driving before it was stopped manually.

Auxiliary task: The auxiliary task was used to set the focus of the feature extractor on traffic lights during training, but this output was not used during testing. Table 1 shows the AVR results of the comparison of the multi-task model with (Model A) and without (Model E) the auxiliary task; a lower AVR is better. The model with the auxiliary task violated one out of three red traffic lights. Without the prediction of the traffic light state, the vehicle violated nearly two out of three traffic lights, an increase of almost 100%. The overall driving performances of both models were comparable, which is reflected by the similar MTTF scores (Model A had 109 s and 84 s, while Model E had 313 s and 78 s for training and the new environment, respectively). The lower MTTF of Model A in the training environment was induced by casual confusion, since this model performed more full stops. These results show that the network learned to focus on traffic lights without significantly losing driving performance.

**Figure 9:**
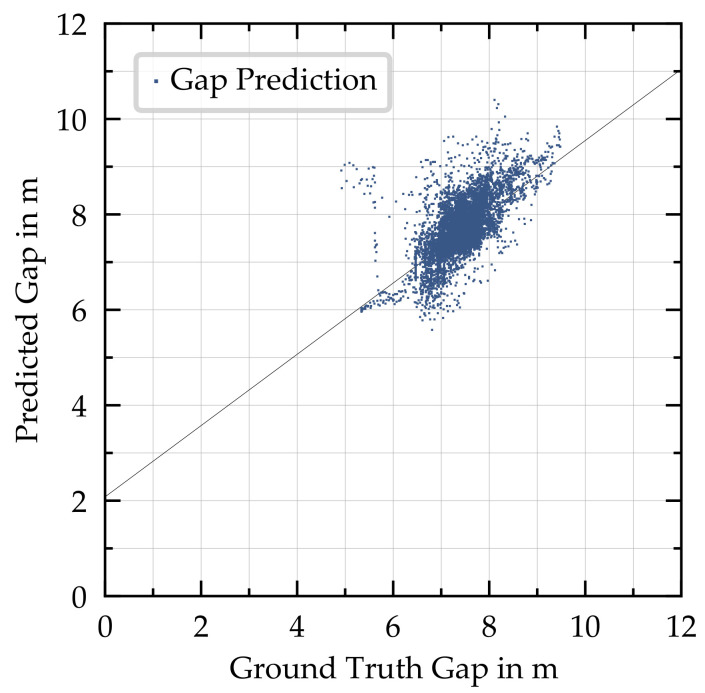
Accuracy of the gap prediction. Every point is a gap prediction captured on a single route, with 14,400 predictions in total. The solid black line indicates the linear regression of all points.

Gap estimation: For the following vehicle, we predicted the gap to the leader instead of throttle/brake commands to avoid casual confusion. Figure 9 shows the accuracy of the gap prediction. Each predicted gap was compared with the ground-truth gap at the time of prediction. All points were captured on a single route containing 14,400 predictions in total, with the desired gap set to 7.5 m. This route included multiple poses of the leading vehicle and situations in which only parts of the leading vehicle were visible to the following vehicle due to the curvature of the road. The RMSE of the prediction on this route was 0.44 m, and the $R^{2}$ was 0.532. In general, a higher ground-truth gap led to a higher prediction, with a few exceptions.

## 6. Discussion

The results show that networks trained in an end-to-end fashion can be used to drive a CAV platoon with two vehicles. In our experiments, the performance of the leading vehicle, in particular, improved in comparison with single-task networks, which was demonstrated by the fact that the ACR more than doubled in both environments. If the following vehicle lost track of the leader, e.g., during sharp turns, the vehicle could benefit from the knowledge it had gained through the joint training. If the following vehicle separates from the platoon, it could simply switch the prediction head and therefore change its state to act as a leader.

For the following vehicle, we achieved an MTTF of 895 s with the multi-task network baseline (Model A) in the training environment. Reference [34] reported an MTTF of 58 s for a following vehicle in a simulation with their best model. Although the results are not directly comparable, since we used a different simulation, our higher MTTF is still a significant improvement and may be attributed to the joint training of our multi-task network and to our model’s use of V2V communication.

In contrast to [32], the overall performance of the multi-task network with LSTM extension (Model B) was the lowest among the evaluated models. We used a single set of sequence lengths and sampling intervals (three each). However, an extensive study of these parameters could improve the performance. Tuning of other hyperparameters such as the number of LSTM layers, the number of neurons per layer, and the position of the LSTM layers within the network could also lead to a performance increase. In addition, we observed that Model B tended to oscillate within the lane, which may lower the riding comfort for passengers. It is not known if the LSTM model of [32] is also prone to oscillations, since their offline evaluation was limited to a dataset in which the control command of the current time step has no influence on the vehicle’s position in the next time step.

The results of the multi-task network with a pre-trained feature extractor (Model C) varied for the leading and following vehicle. This model achieved the best performance when used in the following vehicle, but used in the leading vehicle, it was outperformed by the multi-task network baseline using PilotNet as a feature extractor with fewer convolutional layers. The leading vehicle drove in a stable manner, but did not always obey the given high-level command, which led to lower performance in both the ACR and MTTF.

The performance of the single-task networks (Model D) is significantly worse than the performance of the multi-task network (Model A) in both conducted experiments. Since other parameters of the models are identical, this performance difference can be attributed to multi-task learning. This proves the initial assumption that multi-task learning can improve the platoon’s performance.

The auxiliary task for the traffic light prediction proved able to raise the network’s awareness for traffic lights while maintaining the driving performance. Without the auxiliary task (Model E), the average violation rate of red traffic lights increased by about 100% compared to the model with the auxiliary task (Model A). However, Model A still violated one-third of all red traffic lights. Further investigations have be done to improve driving behavior with respect to traffic lights, since this is not only limited to platooning, but a general challenge for all end-to-end self-driving approaches.

The longitudinal control of the following vehicle was based on information transmitted via V2V communication and the predicted gap. This structure was not fully end-to-end, but it solved the casual confusion for the following vehicle, whereas the casual confusion still existed for the leading vehicle and lowered its performance significantly. Reference [47] suggested including an auxiliary task that predicts the ego velocity to solve the problem of casual confusion. However, even though the authors noticed a performance increase, the casual-confusion problem was still not solved.

Although the latency of V2V communication in our simulation is nonexistent, real-world applications of V2V communication following the IEEE 802.11p standard with an update rate of 10 Hz show a distance-dependent latency. Since we are using data from the previous time step to predict the next control commands as described in Section 4.2, the transmission of the data via V2V communication must be completed within one interval of the update rate, which is 100 ms for our model. Therefore, any latency below 100 ms has no impact on the computed control commands of our model. For a close range (<35 m between the vehicles), Reference [48] measured an average latency of less than 50 ms. Based on these measurements, we assume that the latency, including all delays, is always below 100 ms. In case a message is not received because of wireless dropout, the following vehicle could switch to the leader mode at any time.

In this work, we focused on using the camera as the only sensor. Since the camera is not able to measure the gap to the preceding vehicle and its velocity directly, we predict the gap within the neural network and send the velocity information via V2V communication. The accuracy of the distance prediction (RMSE 0.44 m) is sufficient for the downstream car-following model to follow the leading vehicle at a safe distance (7.5 m in the experiments). Using an additional sensor such as LiDAR or radar could yield the gap directly and substitute or support the prediction.

As this work introduces the multi-task network for CAV platoons and is targeted to prove the model’s general ability to drive a CAV platoon, we limit our experiments to a platoon consisting of two vehicles, i.e., one leading and one following vehicle. Another direction for future work could be the extension of this model and the experiments to cover a vehicle platoon with more than two vehicles.

## 7. Conclusions

This paper presents a new multi-task end-to-end self-driving architecture for the special use case of CAV platoons. The architecture consists of a CNN and two fully connected subsequent prediction heads. The CNN serves as a feature extractor to process images captured by a front-facing camera. Only one prediction head is activated at a time, depending on where the network is used—in the leading or following vehicle of the platoon.

Our experiments show that neural networks trained in an end-to-end fashion are capable of driving two vehicles in a platoon autonomously. In particular, we proved that joint training of a network with two similar tasks can increase the overall performance. We modified our architecture to include different concepts proposed in the literature, such as the incorporation of temporal dependencies by using LSTM layers and transfer learning with a pre-trained network. In our application, LSTM layers were not shown to improve the performance. However, transfer learning had a positive impact on the performance of the following vehicle, although the performance of the leading vehicle suffered as it sometimes ignored the high-level navigational command. Adding an auxiliary task, in our case the prediction of the traffic light state, can assist the network to focus on certain features of the images.

In the experiments performed in the simulation, the following vehicle completed most of the routes or achieved high completion rates. The problem of the casual confusion could be solved for the following vehicle by incorporating V2V communication and predicting the gap instead of throttle/brake values. The main limitation of the platoon is the casual confusion of the leading vehicle, which resulted in no acceleration after full stops. Further research could therefore be undertaken to investigate possible solutions to this limitation.

## Figures and Tables

**Table 1:** Average violation rate (AVR) of red traffic lights in % for the multi-task network with (Model A) and without (Model E) the auxiliary task.

	Training Environment		New Environment	
	Model A	Model E	Model A	Model E
AVR	**33**	64	**34**	65
